# Radiological biomarkers reflecting visceral fat distribution help distinguish inflammatory bowel disease subtypes: a multicenter cross-sectional study

**DOI:** 10.1186/s13244-024-01640-9

**Published:** 2024-03-13

**Authors:** Ziman Xiong, Peili Wu, Yan Zhang, Jun Chen, Yaqi Shen, Ihab Kamel, Bing Wu, Xianying Zheng, Zhen Li

**Affiliations:** 1 Department of Radiology, Tongji Hospital, Tongji Medical College, Huazhong University of Science and Technology, 1095 Jiefang Avenue, Qiaokou District, Wuhan, 430030 Hubei China; 2 Department of Radiology, The First Affiliated Hospital of Fujian Medical University, Fuzhou, 350005 Fujian China; 3 Department of Radiology, West China Hospital, Sichuan University, No. 37, Guoxue Alley, Chengdu, 610041 Sichuan China; 4 GE Healthcare, Wuhan, 430030 Hubei China; 5 Department of Radiology, University of Colorado Denver Anschutz Medical Campus, Aurora, MD 80045 USA; 6 Department of Radiology, Fujian Maternity and Child Health Hospital, Fuzhou, 350005 Fujian China

**Keywords:** Inflammatory bowel disease, Computed tomography, Visceral adipose tissue, Diagnosis, Biomarker

## Abstract

**Objectives:**

To achieve automated quantification of visceral adipose tissue (VAT) distribution in CT images and screen out parameters with discriminative value for inflammatory bowel disease (IBD) subtypes.

**Methods:**

This retrospective multicenter study included Crohn’s disease (CD) and ulcerative colitis (UC) patients from three institutions between 2012 and 2021, with patients with acute appendicitis as controls. An automatic VAT segmentation algorithm was developed using abdominal CT scans. The VAT volume, as well as the coefficient of variation (CV) of areas within the lumbar region, was calculated. Binary logistic regression and receiver operating characteristic analysis was performed to evaluate the potential of indicators to distinguish between IBD subtypes.

**Results:**

The study included 772 patients (365 CDs, median age [inter-quartile range] = 31.0. (25.0, 42.0) years, 255 males; 241 UCs, 46.0 (34.0, 55.5) years, 138 males; 166 controls, 40.0 (29.0, 53.0) years, 80 males). CD patients had lower VAT volume (CD = 1584.95 ± 1128.31 cm^3^, UC = 1855.30 ± 1326.12 cm^3^, controls = 2470.91 ± 1646.42 cm^3^) but a higher CV (CD = 29.42 ± 15.54 %, *p* = 0.006 and *p* ˂ 0.001) compared to UC and controls (25.69 ± 12.61 % vs. 23.42 ± 15.62 %, *p* = 0.11). Multivariate analysis showed CV was a significant predictor for CD (odds ratio = 6.05 (1.17, 31.12), *p* = 0.03). The inclusion of CV improved diagnostic efficiency (AUC = 0.811 (0.774, 0.844) vs. 0.803 (0.766, 0.836), *p* = 0.08).

**Conclusion:**

CT-based VAT distribution can serve as a potential biomarker for distinguishing IBD subtypes.

**Critical relevance statement:**

Visceral fat distribution features extracted from CT images using an automated segmentation algorithm (1.14 min) show differences between Crohn’s disease and ulcerative colitis and are promising for practical radiological screening.

**Key points:**

• Radiological parameters reflecting visceral fat distribution were extracted for the discrimination of Crohn’s disease (CD) and ulcerative colitis (UC).

• In CD, visceral fat was concentrated in the lower lumbar vertebrae, and the coefficient of variation was a significant predictor (OR = 6.05 (1.17, 31.12), *p* = 0.03).

• The differences between CD, UC, and controls are promising for practical radiological screening.

**Graphical Abstract:**

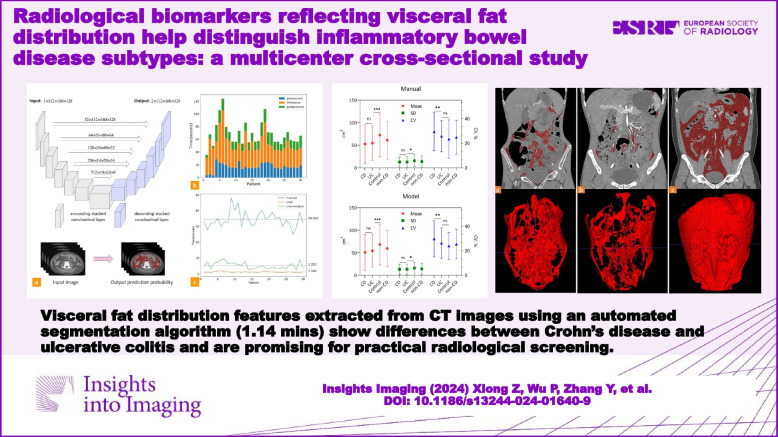

**Supplementary Information:**

The online version contains supplementary material available at 10.1186/s13244-024-01640-9.

## Introduction

Ulcerative colitis (UC) and Crohn’s disease (CD) are two forms of inflammatory bowel disease (IBD). Compared to UC, where the lesions only involve the mucosa and submucosa, lesions in the CD are penetrating and patients are more prone to complications such as intestinal obstruction and inter-intestinal fistula, leading to a higher risk of surgery [1]. Moreover, CD patients often require more systematic and complex drug therapy. Current guidelines emphasize differentiating IBD through a comprehensive assessment of clinical, endoscopic, pathological, and imaging data [2]. Nonetheless, the clinical symptoms in CD and UC patients often overlap. As a feature of CD, the positive pathology rate of non-caseating granulomas is low [3]. In addition, endoscopy and radiology have limited ability to depict transmural inflammation in CD [4]. Consequently, distinguishing between CD and UC can be challenging, especially in patients with only colonic lesions [5].

Visceral adipose tissue (VAT) in IBD is recognized as an endocrine organ associated with disease progression and adverse outcomes, rather than an inert energy-storing tissue [6]. Particularly in CD, the creeping fat surrounding the affected intestinal segment is closely associated with complex disease phenotype [7, 8]. Previous studies have observed higher VAT content in UC compared to CD patients [9]. Researchers have also investigated the difference in VAT area (VFA) and the ratio of VFA to the subcutaneous fat area (VSR) at the third lumbar vertebrae (L3) level on CT images in CD, UC, and control populations [10]. However, due to the individualized differences in nutritional status, and the fact that accumulation of adipose tissue in CD is usually related to the diseased intestinal segment, using indicators obtained from single-slice images for differential diagnosis can be challenging [5, 11]. Evaluation of whole abdominal CT is currently emerging as the most accurate method to provide precise measurement of VAT volume [12], and abdominal CT scans are routinely performed in IBD populations due to the need for diagnosis and follow-up [13]. Automated VAT volume quantification on CT can be achieved using deep learning models, which is helpful for use in large-scale populations [14]. In addition, algorithms can also quantify the VAT distribution based on anatomical location, thus revealing the relationship between different cross-sectional areas and volumes. Studies have been conducted to analyze the distribution of VAT in overweight or obese patients [15], but no research has yet extended this method to the IBD population. Therefore, this study aimed to investigate the distribution characteristics of VAT with the assistance of a deep learning model in IBD subjects, to distinguish IBD subtypes.

## Materials and methods

### Study participants

This multicenter retrospective study conducted at three institutions was approved by the Research Ethics Committees from each of the participating centers, and informed consent was waived. Researchers reviewed and identified patients who underwent abdominal CT scans and were diagnosed with CD or UC between 2012 and 2021 by searching the electronic medical record system. The diagnosis was confirmed under the World Gastroenterology Organization global guidelines [2]. The following patients were excluded: (1) age < 18 years old, (2) CT data were unavailable, (3) patients had comorbidities including cancer or renal failure, (4) patients received immunotherapy for other reasons within 6 months, (5) underwent prior intestinal surgery. The scan closest to the initial diagnosis was selected in patients with multiple CT scans.

Referring to previous studies [10], the control group consisted of patients admitted between 2012 and 2021 with acute appendicitis, who were in good health prior to the onset of acute abdominal pain, and underwent abdominal CT scans before surgery. The patient’s sex, age, height, weight, and smoking history were extracted from the electronic medical record, as well as Montreal classification [16], laboratory indicators (serum albumin (Alb); C-reactive protein (CRP); erythrocyte sedimentation rate (ESR)), and surgical records within the following 6 months. The body mass index (BMI) was obtained by dividing the weight by the square of the height and 24 kg/m^2^ was used as the cut-off point for overweight according to a prior study [17].

### Development of an automatic VAT segmentation model

#### Semi-automatic segmentation of VAT in CT images

Across different time periods and geographic regions, abdominal CT protocols have displayed variations, yet all have consistently surpassed the essential technical prerequisites [18]. For IBD patients undergoing CT scans in the outpatient department, they were required to fast for 4 to 6 h before the scan and avoid gas-producing liquids. Additionally, they were instructed to ingest 1000 to 1500 mL of aqueous 2.5% mannitol within 45 to 60 min before the scan. For patients with IBD and appendicitis undergoing CT scans in the emergency department, the above preparation was omitted and they proceeded directly to enhanced CT scanning. All patients started with a pre-contrast scan, and then, contrast-enhanced CT was performed after a rapid bolus of iopromide (Ultravist 370, 370 mg/ mL, Bayer Schering Pharma, Berlin, Germany) (1.5 mL/kg) at a rate of 3–5 mL/s, followed by a 20-mL saline flush using a power injector. Images were routinely obtained in the arterial, intestinal, or portal venous phases. All CT scans covered the whole abdomen and pelvic cavity, and the maximal slice thickness was 3 mm. Arterial phase images were selected for further analysis. A semi-automated method was used to quantify the VAT between the dome of the diaphragm and the pubic symphysis on the patient’s CT image [19]; the procedure was described in the supplementary methods.

#### Construction of an automatic segmentation model

We constructed an automatic VAT segmentation algorithm based on a 3D U-shape convolutional neural network (CNN), and Table S1 and S2 show detailed information. We trained and validated our segmentation model using the fivefold cross-validation algorithm in center 1. Data from the other two centers were deployed to validate the generalization of the model. To assess the model’s reliability and effectiveness, we conducted a test wherein 30 patients were randomly selected from 3 different centers. Subsequently, we utilized both semi-automatic and automatic methods to delineate VAT regions. The time taken for each approach was recorded for further analysis. The segmentation results were compared with the previous semi-automatic results, and the Dice and Jaccard were calculated. Furthermore, elapsed time comparison was conducted between semi-automated segmentation, unet, and unet+ adjustment.

#### Extraction of VAT indicators

The VAT volume was quantified using the masks generated from the semi-automated and automated methods. Multiple 2D axial slices from each patient’s CT images were selected for analysis, with detailed steps described in the supplementary methods, and representative 35 slices were selected from the first to the fifth lumbar vertebra and pelvic level. The area of the selected image is automatically calculated through the marked layer. Then, the measured lumbar height (vertical height between L1-1 and L5-5) was used to standardize VFA as follows: standardized index (visceral adipose index, VAI) = VFA/height_L1-L5_^2^ (cm^2^/m^2^) [20]. We assessed the VAT distribution by calculating the VAT ratio at each level as follows: VAT ratio (%) = (VFA × layer thickness)/VAT volume × 100%. The VFAs of all slices in the lumbar region (L1 to L5) were calculated, and the mean, standard deviation (SD), and coefficient of variation (CV) of areas were extracted to reflect the distribution. The 316 IBD patients in this study were reported previously [19]. The prior report studied the value of radiomics in the identification of IBD subtypes. The current study included more cases, faster analysis methods, and more interpretable quantitative parameters to extend this finding.

### Statistical analysis

Quantification of VFA, volume, and distribution was performed using Python version 3.7, and the source code is available at https://github.com/CharelBIT/nnUNet-modify. Statistical analyses were performed using SPSS version 26.0 and MedCalc Statistical Software version 20.100. Comparisons between two groups of continuous variables were made using Student’s *t*-test or Mann-Whitney U-test, and comparisons between the three groups of continuous variables were made using one-way ANOVA or Kruskal-Wallis test as appropriate. The *χ*^2^ test was used to compare categorical variables. The Pearson/Spearman correlation coefficient was used to analyze the correlation between variables. Binary logistic regression and receiver operating characteristic (ROC) analysis were performed to evaluate the potential of indicators to distinguish between CD and UC patients, and the area under the ROC curve (AUC) was compared between semi-automatic and automatic ways using the DeLong method. Statistical significance levels were set at *p* < 0.05 for two groups, and 0.0167 (corrected *p* = 0.05/3 = 0.0167) for three groups.

## Results

### Clinical characteristics of patients

**Fig. 1:**
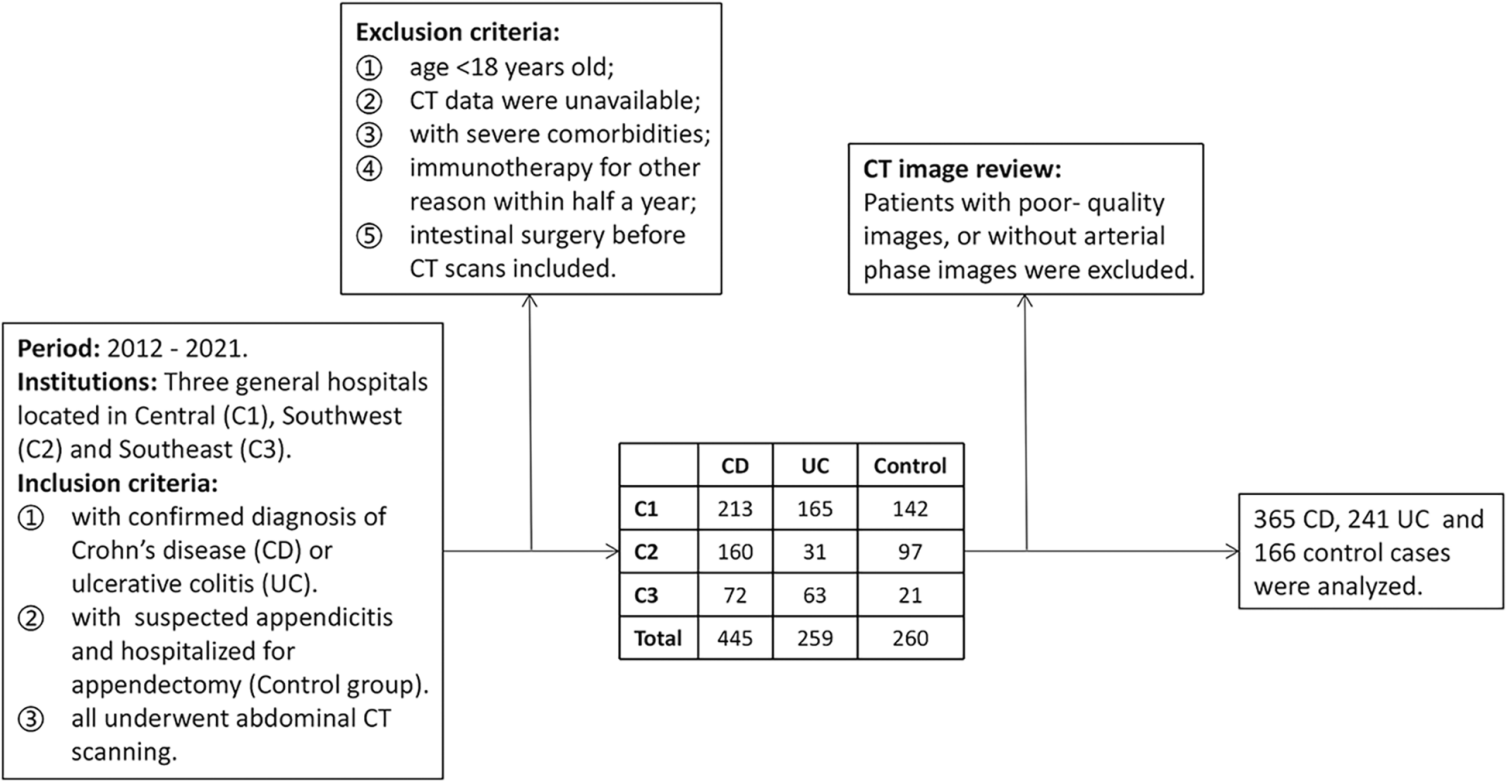
Inclusion flowchart of study subjects

A total of 772 patients (365 CD patients, 241 UC patients, and 166 controls) were included. Figure 1 shows the detailed steps. Comparisons among CD, UC, and controls are shown in Table 1. Compared to the other two groups, the CD group had more males and was generally younger but had a lower BMI (half were underweight). The prevalence of perianal disease was 37.5% (129/344) in the CD group compared to only 7.6% (18/237) in the UC group. In terms of laboratory indicators, Alb levels were generally low in IBD patients; CRP and ESR levels were significantly higher in CD than in UC (*p* < 0.001). In addition, 16.7% (61/365) of CD patients had lesions involving only the colon. At follow-up, a significantly higher proportion of CD patients had undergone bowel resection within 6 months (24.7% vs. 5.9%).

**Table 1:** Comparison of clinical characteristics of all patients

	**CD (*n* = 365)**	**UC (*n* = 241)**	**Control (*n* = 166)**	***p***	**P1**^a^	**P2**^a^	**P3**^a^
**Sex (male/female)**	255/110	138/103	80/86	˂ 0.001	˂ 0.001	˂ 0.001	0.09
**Age (years)**	31.0 (25.0, 42.0)	46.0 (34.0, 55.5)	40.0 (29.0, 53.0)	˂ 0.001	˂ 0.001	˂ 0.001	0.02
**Height (m)**	1.68 (1.60, 1.72)	1.65 (1.69, 1.70)	1.64 (1.59, 1.71)	0.08	0.08	0.06	0.72
**Weight (kg)**	51.8 (46.0, 59.0)	58.7 ± 10.6	57.9 (50.3, 65.0)	˂ 0.001	˂ 0.001	˂ 0.001	> 0.99
**Body mass index (kg/m**^**2**^**)**	*n* = 272	*n* = 142	*n* = 99	˂ 0.001	˂ 0.001	˂ 0.001	0.10
*Underweight (< 18.5)*	129 (47.4%)	29 (20.4%)	11 (11.1%)				
*Normal (18.5–23.9)*	125 (46.0%)	83 (58.5%)	70 (70.7%)				
*Overweight (≥ 24)*	18 (6.6%)	30 (21.1%)	18 (18.2%)				
**Active smoker (yes/no)**	37/312	27/211	25/136	0.27	0.78	0.15	0.29
**Disease duration (years)**	*n* = 349	*n* = 237		-	0.19	-	-
*Median(IQR)*	2 (0.50, 5.0)	2 (0.54, 6.0)	-				
*Range*	0.006–30	0.006–20	-				
**Perianal disease (yes/no)**	129/215	18/219	-	-	˂ 0.001	-	-
**Laboratory findings**							
*Alb (g/L)*	37.90 (32.25, 41.60)	39.7 (34.0, 43.5)	-	-	0.003	-	-
*CRP (mg/L)*	22.10 (6.42, 55.78)	6.7 (1.8, 21.3)	-	-	˂ 0.001	-	-
*ESR (mm/hr)*	27 (10, 52)	16.0 (7.0, 38.0)	-	-	˂ 0.001	-	-
**Disease location**							
**CD**							
*Ileal*	99 (27.1%)	-	-	-	-	-	-
*Colonic*	61 (16.7%)	-	-	-	-	-	-
*Ileocolonic*	205 (56.2%)	-	-	-	-	-	-
*Upper digestive tract*	26 (7.1%)	-	-	-	-	-	
**UC**							
*Ulcerative proctitis*	-	35 (14.5%)	-				
*Left-sided*	-	78 (32.4%)	-				
*Extensive*	-	128 (53.1%)	-				
**Follow-up**	*n* = 344	*n* = 237					
Intestinal resection (yes/no)	85/259	14/223	166/0	-	˂ 0.001	-	-

*CD* Crohn’s disease, *UC* Ulcerative colitis, *IQR* Interquartile range, *Alb* Serum albumin, *CRP* C-reaction protein, *ESR* Erythrocyte sedimentation rate

^a^P1, CD group and UC group; P2, CD group and control group; P3, UC group and control group

### Performance of automatic VAT segmentation models

**Fig. 2:**
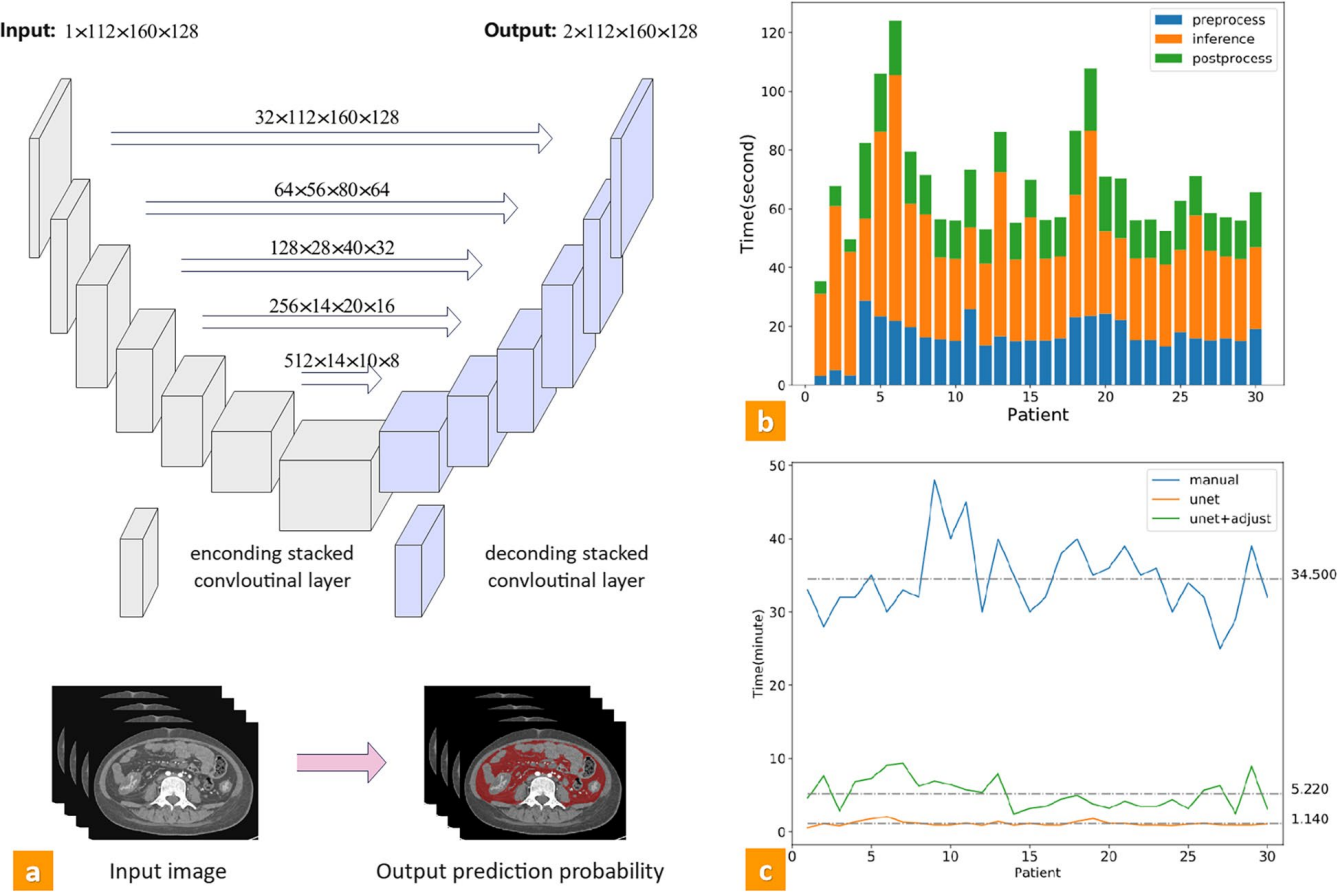
Construction and efficiency testing of automatic segmentation algorithm. **a** The topology of the whole network consists of the encoding and decoding parts. There are five stages in the encoding and decoding subnetwork, indicating that five-level scales of feature maps were formulated for automatic feature extraction. **b** The time required by the automatic segmentation algorithm, including preprocessing, inference, and post-processing. **c** The time required by the semi-automated method, U-net model, and U-net model plus subsequent adjustments

The network topology of the automatic segmentation algorithm is shown in Fig. 2a. The Dice scores were above 0.90 for the training set and above 0.85 for all the testing and validation sets (Table S3). The VAT volumes obtained from the semi-automatic and automated processes were highly correlated (*r* = 0.99, *p* < 0.001). The results of the repeatability test are shown in Table S4. The automatic model took an average of 3.3% (1.14/34.50 min) of the time required by the semi-automatic method to complete the segmentation of a patient. Subsequent adjustments resulted in an improvement of the model’s Dice score, and the automatic method plus manual adjustment process took 15.1% (5.22/34.50 min) of the time of the semi-automatic methods (Fig. 2b, c).

### Comparison of the VAT characteristics among the three groups

**Fig. 3:**
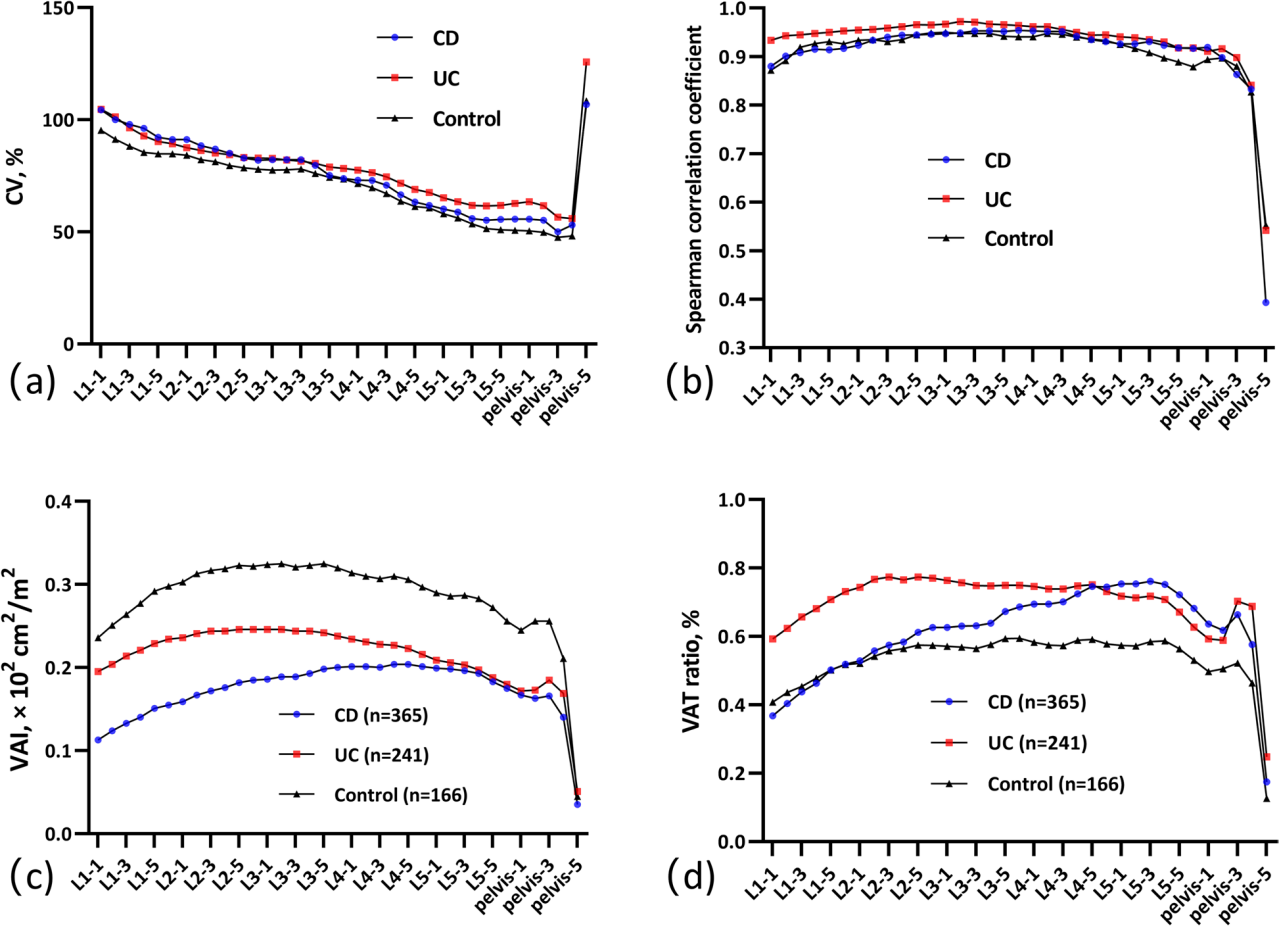
Scatter plots describing various visceral adipose tissue indicators in CD, UC, and control patients. **a** Inter-subject CV of VFA. **b** Correlation between VFA and VAT volume. **c** VAI. **d** VAT ratio. VAT, visceral adipose tissue; CD, Crohn’s disease; UC, ulcerative colitis; CV, coefficient of variation; VFA, visceral fat area; VAI, visceral adipose index

The volume of VAT was significantly lower in CD (1584.95 ± 1128.31 cm^3^) and UC patients (1855.30 ± 1326.12 cm^3^) than in controls (2470.91 ± 1646.42 cm^3^, *p* < 0.001). The intra-subject CVs across 35 slices were 34.08 ± 11.14% in CD, 31.08 ± 9.93% in UC, and 29.92 ± 8.97% in controls, respectively, and the CV in CD was significantly higher (*p* < 0.05). The CVs of VFA at each analyzed level of the three groups are shown in Fig. 3a, and the CVs decreased with decreasing vertebral level (except for Pelvis-5) and the trend was consistent among the three groups. The correlation between VFA and VAT volume at each analyzed level of the three groups is shown in Fig. 3b. Correlation coefficients at all levels were greater than 0.80 except Pelvis-5 (*p* < 0.01), with the strongest correlation level being L3-L4 in CD (*r* = 0.954), the upper part of L3 in UC (*r* = 0.972), and controls (*r* = 0.950). Comparisons of VAI at different levels among the three groups are shown in Fig. 3c (Table S5), and the VAIs were generally lower in IBD cases. Comparisons of the VAT ratio are shown in Fig. 3d (Table S6). The trend in UC resembled that of the control group, displaying a relatively even pattern. Conversely, CD showed a concentration primarily in the lower lumbar region. We also described the trends according to sex, as shown in Figure S3b, where the trends of UC and controls were still similar and distinguished from CD.

### Differences in VAT distribution among the three groups

**Fig. 4:**
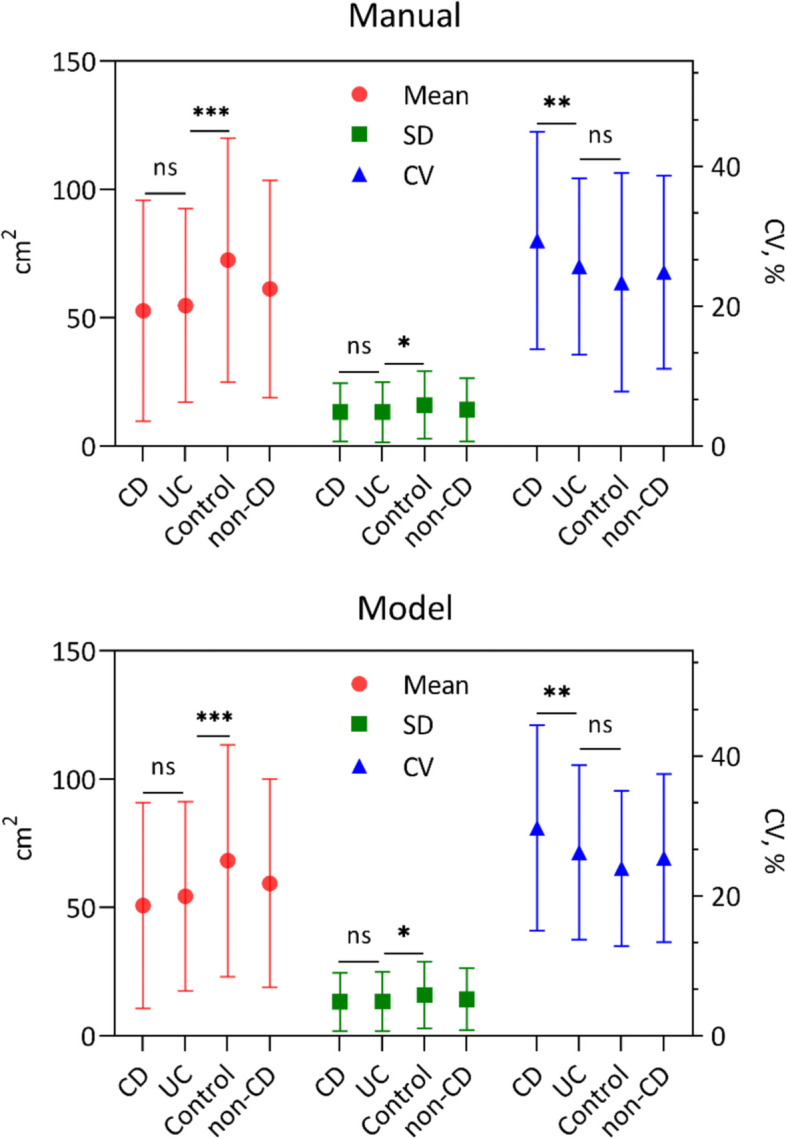
Boxplots of VAT distribution indexes within the lumbar region in three groups. The differences among groups of each index calculated by semi-automatic and automatic segmentation results were consistent. VAT, visceral adipose tissue; CD, Crohn’s disease; UC, ulcerative colitis; SD, standard deviation; CV, coefficient of variability, *p < 0.05, **p < 0.01, ***p < 0.001

**Fig. 5:**
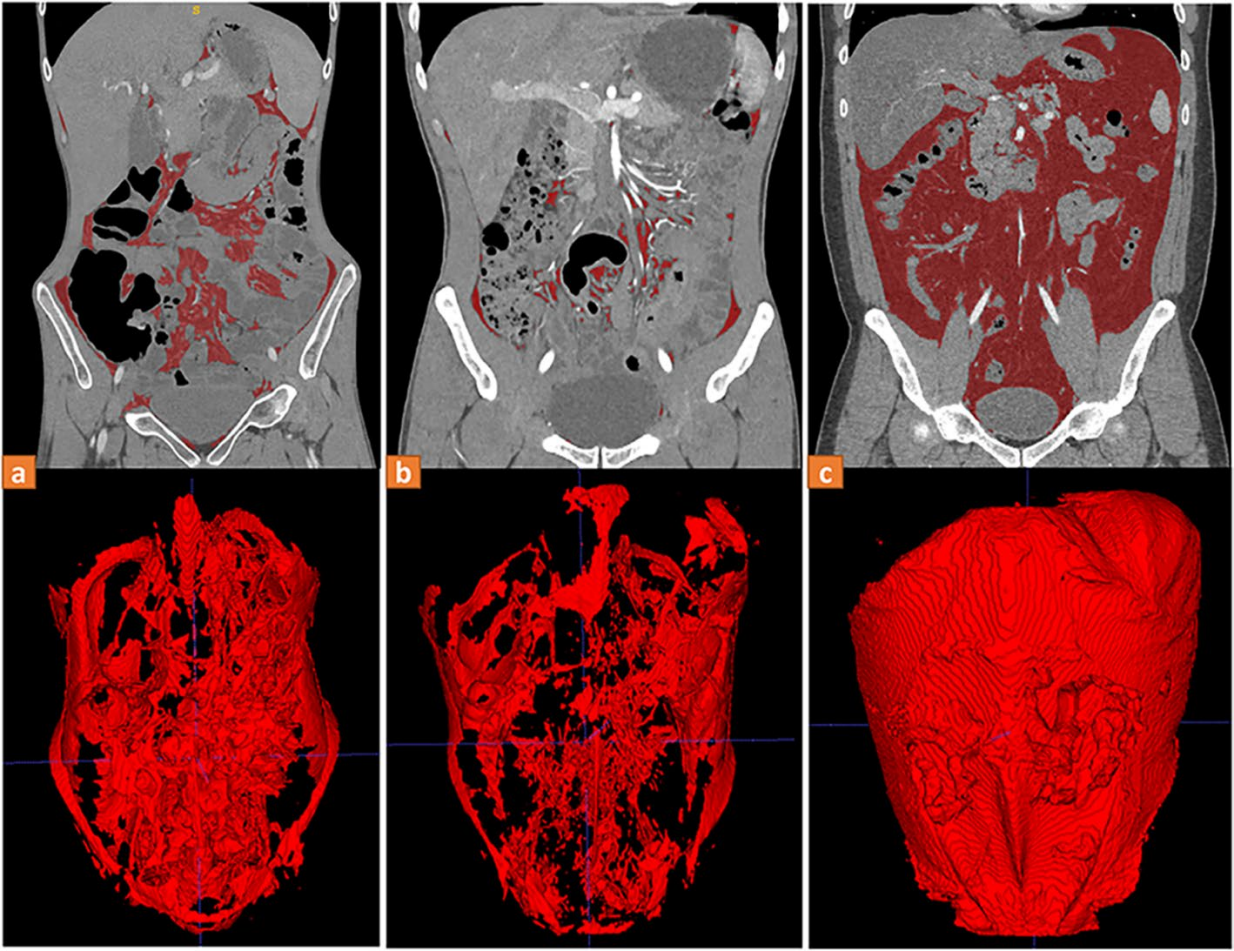
Coronal CT images of three patients diagnosed with CD, UC, and acute appendicitis, and their visceral adipose tissue was marked red. **a** A 24-year-old male CD patient with a BMI of 12.35 kg/m^2^ presenting with ileocolonic CD. The average VFA within the lumbar region was 22.46 cm^2^ with a CV of 66.39%. **b** A 30-year-old male UC patient with a BMI of 17.82kg/m^2^, presenting with mild UC. The average VFA was 58.84 cm^2^, and the CV was 30.15%. **c** A 32-year-old male appendicitis patient had a BMI of 27.71kg/m^2^. The average VFA was 161.30 cm^2^, and the CV was 28.86%. CD, Crohn’s disease; UC, ulcerative colitis; BMI, body mass index; VFA, visceral fat area; CV, coefficient of variation

The indicators reflecting VAT distribution are summarized in Table 2 (Fig. 4). In the mean value and SD, the CD and UC groups were similar, and lower than the controls, while the CD group had the largest CV reflecting the more heterogeneous VAT distribution within the lumbar region of CD patients. The difference in CV between UC and controls was not significant, although UC had a lower mean and SD. The above comparison results were consistent whether using semi-automated or automatic segmentation results. In addition, we compared the VAT distribution between UC patients with and without perianal fistula and found no significant differences in CV between them (0.27 (0.19, 0.37) vs. 0.24 (0.17, 0.31), *p* = 0.31). Figure 5 shows coronal CT images of three patients diagnosed with CD, UC, and acute appendicitis. The VAT of the CD patients was significantly more concentrated in the lower lumbar region, while the distribution of VAT in the UC and the control group was more uniform and similar. Furthermore, binary logistic regression showed that after adjusting clinical indicators, CV was still a predictor of CD (OR = 6.05 (1.17, 31.12), *p* = 0.03) (Table S7). ROC analysis demonstrated that the diagnostic efficacy of the automatic model was comparable to that of semi-automatic techniques (AUC = 0.810 (0.773, 0.843) vs. AUC = 0.811 (0.774, 0.844), *p* = 0.38), and improved the efficiency of clinical indicators (AUC = 0.803 (0.766, 0.836), *p* = 0.10 and 0.08) (Table 3).

**Table 2:** Comparison of the mean, standard deviation, and coefficient of variability of visceral fat areas within the lumbar region

	**CD (*n* = 365)**	**UC (*n* = 241)**	**Control (*n* = 166)**	**P1**^a^	**P2**^a^	**P3**^a^
**Mean (cm**^**2**^**)**						
*Semi-automatic*	52.80 ± 42.99	54.83 ± 37.69	72.53 ± 47.55	0.80	˂ 0.001	˂ 0.001
*Automatic*	50.79 ± 40.16	54.47 ± 36.87	68.26 ± 45.18	0.44	˂ 0.001	0.002
**SD (cm**^**2**^**)**						
*Semi-automatic*	13.25 ± 11.27	13.25 ± 11.73	16.12 ± 13.14	> 0.99	0.01	0.04
*Automatic*	13.25 ± 11.29	13.42 ± 11.47	15.92 ± 12.97	0.86	0.02	0.04
**CV (%)**						
*Semi-automatic*	29.42 ± 15.54	25.69 ± 12.61	23.42 ± 15.62	0.006	˂ 0.001	0.11
*Automatic*	29.73 ± 14.67	26.23 ± 12.47	23.96 ± 11.09	0.007	˂ 0.001	0.08

*CD* Crohn’s disease, *UC* Ulcerative colitis, *SD* Standard deviation, *CV* Coefficient of variability

^a^P1, CD and UC; P2, CD and control; P3, UC and control

**Table 3:** Comparison between areas under the receiver operating characteristic curve for differentiation between Crohn’s disease and ulcerative colitis groups

	**Age**	**Perianal disease**	**CRP**	**ESR**	**Clinical combination**	**CV**		**Combine all**	
						**Semi-automatic**	**Automatic**	**Semi-automatic**	**Automatic**
**Sensitivity (%)**	66.58 [61.5, 71.4]	37.50 [32.4, 42.9]	66.96 [61.7, 72.0]	65.75 [60.3, 70.9]	70.32 [64.9, 75.4]	41.60 [35.3, 48.1]	28.18 [23.6, 33.1]	70.36 [64.9, 75.4]	65.15 [59.5, 70.5]
**Specificity (%)**	70.95 [64.8, 76.6]	92.41 [88.3, 95.4]	62.33 [55.6, 68.7]	51.80 [45.0, 58.5]	77.36 [71.1, 82.8]	69.61 [64.6, 74.3]	83.61 [78.3, 88.1]	79.43 [73.3, 84.7]	85.65 [80.1, 90.1]
**AUC**	0.732 [0.695, 0.767]	0.650 [0.609, 0.688]	0.670 [0.629, 0.709]	0.599 [0.556, 0.640]	0.803 [0.766, 0.836]	0.568 [0.527, 0.608]	0.571 [0.530, 0.611]	0.810 [0.773, 0.843]	0.811 [0.774, 0.844]
***p*-value**					0.10, 0.08^a^	0.57		0.38	

*AUC* Area under the curve, *CRP* C-reaction protein, *ESR* Erythrocyte sedimentation rate, *CV* Coefficient of variability

^a^Compared with semi-automatic and automatic combinations, respectively

## Discussion

In this study, a deep learning approach was used to achieve automated quantification of VAT, which was used to investigate the VAT distribution in CD, UC, and controls. Unlike the relatively uniform VAT distribution in the UC and controls, the VAT in CD was more concentrated in the lower lumbar vertebrae and further analysis revealed a greater variability of VAT distribution in CD patients. Subsequent analyses also confirmed the ability of CV to distinguish between CD and UC patients and improved diagnostic performance when combined with clinical indicators.

Previous body composition studies conducted in the IBD population mainly used semi-automated quantification methods based on single-slice images [10, 21, 22], and the frequent usages of single-slice areas are possibly due to its simplicity. The development of automated segmentation of body composition has enabled fast and accurate quantification of multi-slice VFAs and VAT volumes [14, 23, 24], which has been achieved in this study. Our developed model exhibited commendable accuracy not only in IBD patients, but also in individuals diagnosed with acute appendicitis. Moreover, its strong performance persisted across different medical imaging machines and healthcare institutions, underscoring its resilience and applicability. In addition, this method took much less time than the semi-automatic method.

Differences in VAT content among CD, UC, and control (acute appendicitis) populations have been compared. Zhang et al. found that compared with UC and controls, the CD group had lower VFA but higher VSR in a single CT image at the L3 level. The difference between UC and controls was not significant [10]. Jahnsen et al. quantified the body composition through dual X-ray absorptiometry and found significantly lower VAT content in CD than in UC [9]. Clinical imaging analyses have also been performed in IBD patients using representative levels such as L3 or L4, revealing the impact of VAT on CD disease phenotype, activity, and prognosis [20, 21, 25]. Although our analysis showed a high correlation between VFA and VAT volume at all lumbar vertebra levels in the IBD groups, it is not a reliable indicator for identifying and assessing IBD, given that VAT content is closely related to individual nutritional status and disease duration. Therefore, in the current study, the vertebral level-VAT ratio curve was described to show the trend of VAT distribution, and the CV of VAT distribution in the lumbar region was also calculated. Our results show that the VAT distribution in CD is concentrated in the lower lumbar region compared to the relatively uniform distribution in non-CD groups. Furthermore, this trend was observed consistently in both male and female patients. Subsequent analysis unveiled that the CV in VAT distribution was notably higher in CD compared to those with UC. Conversely, no discernible difference between UC and controls was found, which was not mentioned previously. Based on these findings, we devised a novel approach for identifying CD and UC individuals by integrating CV measurements with pertinent clinical indicators. Notably, CV emerged as a significant predictor for CD.

Abnormal hyperplastic mesenteric adipose tissue (MAT) can be seen around inflamed intestinal segments in CD patients, which can occur early in the disease [26]. Under normal conditions, MAT participates in the balance of local and systemic immune microenvironments. It serves as a physical barrier to block the spread of inflammation, absorbs excess fat and sugars, and secretes various anti-inflammatory factors thereby reducing the severity of intestinal inflammation [27]. However, for CD patients, MAT is a repository for dysfunctional immune cells, and it secretes more fatty acids and increases immune cells and extracellular matrix, exacerbating intestinal inflammation [28, 29]. Interestingly, some studies have found that VAT may play a different role in UC. A study by Zulian et al. revealed differential inflammatory gene expression as well as bacterial load in the MAT of CD and UC [30], suggesting that VAT plays an important role in the pathophysiology of CD, but not in UC. Therefore, the function of VAT in UC may be closer to that of normal people than that of CD. Considering that the distribution of MAT in CD patients is related to the affected intestinal segment, and CD mainly affects the ileum, this may explain why our results showed a similar VAT distribution between UC and controls, while CD was more concentrated in the lower abdomen.

This multicenter study has some limitations. First, inherent flaws exist in retrospective study design. Due to the considerable time and space spanned, variations in CT scanning protocols, whether within the same or different locales, are practically unavoidable. To address this challenge, we adopted a focused strategy, specifically selecting arterial phase CT images for analysis. Furthermore, to mitigate potential heterogeneity, we normalized both spatial and intensity before feeding them into the neural network for processing. Second, due to the importance of VAT for CD, our model currently exclusively identified VAT and did not encompass other body composition such as subcutaneous fat. But subsequent studies will include more labels if necessary. Finally, considering the trade-offs associated with radiation exposure in enhanced CT scans, our control group was composed of patients with acute appendicitis rather than healthy volunteers. To mitigate potential confounding effects on the results, we focused only on VAT content without considering the attenuation value.

Based on the deep learning algorithm, VAT in CT images can be readily identified, allowing for automated extraction of content, and distribution information. In CD patients, the distribution of VAT is concentrated in the lower lumbar level and exhibits greater heterogeneity compared to non-CD patients. This feature may be a potential biomarker to distinguish CD from UC.

### Supplementary Information

**Additional file 1.** Supplementary methods. Supplementary Figures. Supplementary Tables.

## Data Availability

The datasets used or analyzed during the current study are available from the corresponding author upon reasonable request.

## Abbreviations

Alb: Serum albumin
AUC: Area under the curve
BMI: Body mass index
CD: Crohn’s disease
CRP: C-reactive protein
CT: Computed tomography
CV: Coefficient of variability
ESR: Erythrocyte sedimentation rate
IBD: Inflammatory bowel disease
L3: The third lumbar vertebrae
MAT: Mesenteric adipose tissue
ROC: Receiver operating characteristic
SD: Standard deviation
UC: Ulcerative colitis
VAI: Visceral adipose index
VAT: Visceral adipose tissue
VFA: Visceral fat area
VSR: Ratio of VFA to subcutaneous fat area
