# Supplementary material for: Radiological biomarkers reflecting visceral fat distribution help distinguish inflammatory bowel disease subtypes: a multicenter cross-sectional study
Source: Insights Imaging. 2024 Mar 13;15:70. doi: 10.1186/s13244-024-01640-9 (PMC10933218; doi:10.1186/s13244-024-01640-9)
Supplement: Supplementary file 1 — Additional file 1. Supplementary methods. Supplementary Figures. Supplementary Tables. [file 13244_2024_1640_MOESM1_ESM.pdf]

Radiological Biomarkers Reflecting Visceral Fat Distribution Help Distinguish Inflammatory Bowel Disease Subtypes: A Multicenter Cross-sectional study
ELECTRONIC SUPPLEMENTARY MATERIAL

Semi-automatic segmentation of VAT in CT images

The procedure was performed as follows: first, CT images of all patients (772 in total) were approximately divided into six equal parts and then randomly assigned to six radiologists (2-5 years of abdominal radiology experience) who were blinded to the patient's clinical information; they used the semi-automated threshold-based 3D segmentation module of the open source software (ITK-SNAP 3.8.0, www.itksnap.org) to initially contour the VAT between the dome of the diaphragm and the pubic symphysis and then manually corrected it layer by layer. Finally, the segmentation results outlined by the above six radiologists were checked by an experienced radiologist (11 years of experience in abdominal radiology) to correct any extra or missing pixels (e.g., intermuscular as well as intra-intestinal fatty tissue), and a mask of the final VAT was generated for each patient (Figure S1).

The selection process for multiple 2D axial slices

A 3-step method was utilized to achieve uniform slice selection: **(a)** identifying the upper margin of the sacral vertebra and pubic symphysis, as well as the upper and lower margin of each lumbar vertebra (the first to the fifth lumbar vertebra (L1-L5)) in the axial and sagittal plane; **(b)** extracting five axial slices uniformly from each vertebrae and pelvis level (the upper margin of the sacral vertebra to the upper margin of the pubic symphysis) and one axial slice per vertebral space; **(c)** there would be 35 slices identified per CT scan. The slice selection process was performed by another research assistant, see Figure S2 for details.

Supplementary results

Subgroup comparisons of VAT characteristics according to sex and BMI

Sex differences existed in VAT volume, with males being lower than females in CD (1541.12 ± 1206.68 cm^3 vs. 1686.56 ± 918.39 cm^3, $p = 0.004$), and higher in UC (2111.58 ± 1517.12 cm^3 vs. 1517.70 ± 925.28 cm^3, $p = 0.008$) and controls (3086.58 ± 1890.25 cm^3 vs. 1898.20 ± 1119.14 cm^3, $p < 0.001$). Comparisons of VFA based on BMI and sex are shown in Figure S3a. When BMI was in the normal range, VFA was lower in males than in females in CD ($p < 0.001$), while it was higher in males in UC and controls ($p < 0.001$). When underweight, it was still lower in males in CD and higher in males in controls ($p < 0.001$), while the gender difference in UC was not significant ($p = 0.10$). When overweight, a higher VFA in males was observed in all kinds of patients ($p < 0.001$).

Supplementary figures

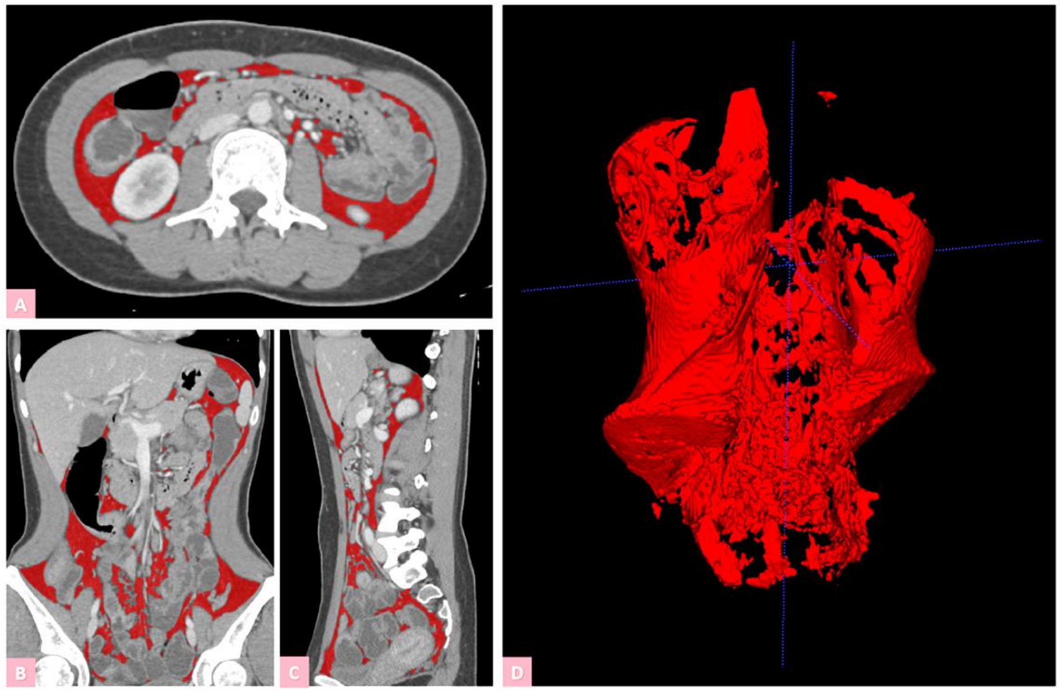

Figure S1 The visceral adipose tissue (VAT) mask generated by a semi-automated method with manual correction.
A, B, and C are the axial, coronal, and sagittal abdominal enhanced CT images of a female patient with Crohn's disease, respectively, where the red area is the identified VAT. D is the generated 3D mask image.

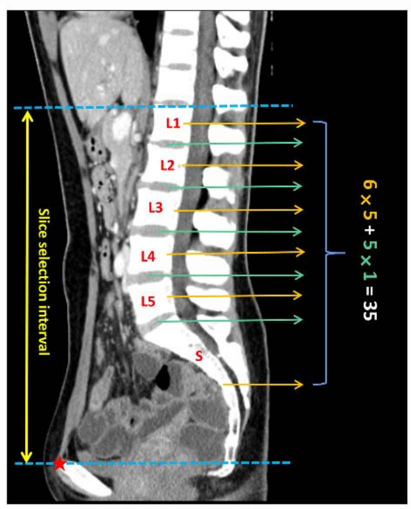

Figure S2 Schematic diagram of the selection of 35 CT image slices.
The blue dashed line indicates the selection interval, and the red star is the upper margin of the pubic symphysis. Five slices were selected evenly within each lumbar vertebrae as well as pelvis level, for a total of 30 slices; one slice was selected in each vertebrae space, for a total of 5 slices.

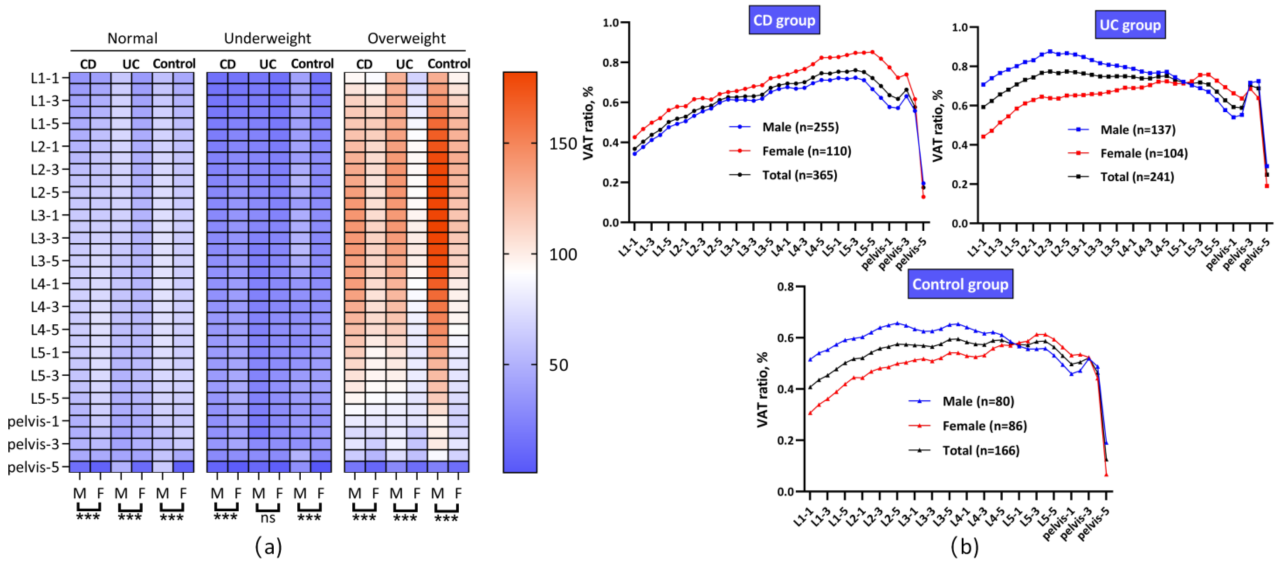

Figure S3 Subgroup comparison of VAT indicators.

(a) Comparisons of VFA based on BMI and sex. The difference in VFA between male and female patients in UC and control groups was similar across all BMI ranges, with higher values in males; while the VFA in the CD group was lower in males in the low to normal BMI range; **(b)** The trends of VAT ratios in CD, UC and control groups with the analyzed levels were described according to sex, where the trends of UC and controls were still very similar and distinguished from CD.

VAT: visceral adipose tissue, VFA: visceral fat area, BMI: body mass index, CD: Crohn’ s disease, UC: ulcerative colitis.

Supplementary tables

Table S1 Details of encoding and decoding subnetwork

Stage	Encoding Sub-network			Decoding Sub-network		
	Input tensor	Output tensor	Basic structure	Input tensor	Output tensor	Basic structure
Stage1	$1 \times 112 \times 160 \times 128$	$32 \times 112 \times 160 \times 128$	$3 \times 3 \times 3$, 32, $1 \times 1 \times 1$ [*CIL*]<br>$3 \times 3 \times 3$, 32, $1 \times 1 \times 1$ [*CIL*]	D2+E1	$2 \times 112 \times 160 \times 128$	*Concat*<br>$3 \times 3 \times 3$, 32, $1 \times 1 \times 1$ [*CIL*]<br>$3 \times 3 \times 3$, 32, $1 \times 1 \times 1$ [*CIL*]<br>$3 \times 3 \times 3$, 2, $1 \times 1 \times 1$ [*C*]
Stage2	$32 \times 112 \times 160 \times 128$	$64 \times 56 \times 80 \times 64$	$3 \times 3 \times 3$, 64, $2 \times 2 \times 2$ [*CIL*]<br>$3 \times 3 \times 3$, 64, $1 \times 1 \times 1$ [*CIL*]	D3+E2	$32 \times 112 \times 160 \times 128$	*Concat*<br>$3 \times 3 \times 3$, 64, $1 \times 1 \times 1$ [*CIL*]<br>$3 \times 3 \times 3$, 64, $1 \times 1 \times 1$ [*CIL*]<br>$2 \times 2 \times 2$, 32, $2 \times 2 \times 2$ [*T*]
Stage3	$64 \times 56 \times 80 \times 64$	$128 \times 28 \times 40 \times 32$	$3 \times 3 \times 3$, 128, $2 \times 2 \times 2$ [*CIL*]<br>$3 \times 3 \times 3$, 128, $1 \times 1 \times 1$ [*CIL*]	D4+E3	$64 \times 56 \times 80 \times 64$	*Concat*<br>$3 \times 3 \times 3$, 128, $1 \times 1 \times 1$ [*CIL*]<br>$3 \times 3 \times 3$, 126, $1 \times 1 \times 1$ [*CIL*]<br>$2 \times 2 \times 2$, 64, $2 \times 2 \times 2$ [*T*]
Stage4	$128 \times 28 \times 40 \times 32$	$256 \times 14 \times 20 \times 16$	$3 \times 3 \times 3$, 256, $2 \times 2 \times 2$ [*CIL*]<br>$3 \times 3 \times 3$, 256, $1 \times 1 \times 1$ [*CIL*]	D5+E4	$128 \times 28 \times 40 \times 32$	*Concat*<br>$3 \times 3 \times 3$, 256, $1 \times 1 \times 1$ [*CIL*]<br>$3 \times 3 \times 3$, 256, $1 \times 1 \times 1$ [*CIL*]<br>$2 \times 2 \times 2$, 128, $2 \times 2 \times 2$ [*T*]
Stage5	$256 \times 14 \times 20 \times 16$	$512 \times 7 \times 10 \times 8$	$3 \times 3 \times 3$, 512, $2 \times 2 \times 2$ [*CIL*]<br>$3 \times 3 \times 3$, 512, $1 \times 1 \times 1$ [*CIL*]	D6+E5	$256 \times 14 \times 20 \times 16$	*Concat*<br>$3 \times 3 \times 3$, 512, $1 \times 1 \times 1$ [*CIL*]<br>$3 \times 3 \times 3$, 512, $1 \times 1 \times 1$ [*CIL*]<br>$2 \times 2 \times 2$, 256, $2 \times 2 \times 2$ [*T*]
Stage6	$512 \times 7 \times 10 \times 8$	$1024 \times 7 \times 5 \times 4$	$3 \times 3 \times 3$, 1024, $2 \times 2 \times 2$ [*CIL*]<br>$3 \times 3 \times 3$, 1024, $1 \times 1 \times 1$ [*CIL*]	E6	$512 \times 7 \times 10 \times 8$	$2 \times 2 \times 2$, 512, $2 \times 2 \times 2$ [*T*]

CIL: Convolutional, Instance Normalization and LeakyReLU layers; T: transposed convolutional layer; Dn: *n*th Decoder part; En: *n*th Encoder part

Table S2 Details of model construction

**Model Construction Details**	**Operations**
CT image preprocess	**Spatial Normalization:**<br>(1) Crop the void area (tissue-free area) in the CT images;<br>(2) Resample the CT images into 1.52*1.52*2.74 (median spacing of C1-central cohort);<br>(3) Randomly crop in the patch size of 112*160*128 to feed into the model.<br>**Intensity Normalization:**<br>(1) Clip the intensity in the 99th percentage of their intensity values;<br>(2) Use the Z-score normalization method.
Data augmentation	**Spatial Data Augmentation:**<br>(1) Elastic deformation;<br>(2) Random Rotation;<br>(3) Random Scale;<br>(4) Mirror Transform<br>**Intensity Data Augmentation:**<br>(1) Gamma Transform;
Details	**Training Strategies:**<br>(1) Deep Supervision;<br>(2) Clip Gradient to avoid divergence；<br>(3) Early Stop if the dice score does not improve in 50 epochs<br>**Loss Function:**<br>Cross Entropy Loss and Dice Loss function was implemented for optimization<br>**Optimizer:**<br>(1) Stochastic Gradient Descent, weight decay: $3*10^{-5}$, momentum: 0.99<br>(2) Poly Learning rate decay method, initial learning rate: 0.01, max epochs: 500<br>**Model Development:**<br>(1) Basic Structure: 3D Convolutional Layer, Leaky ReLU, and Instance Normalization;<br>(2) Kaiming normal distribution for network initialization;<br>**Validation Data Augmentation:**<br>(1) Stride the whole CT images using a fixed patch size;<br>(2) Mirror in the sagittal, axial, and coronal plane;<br>(3) Gaussian Filter to focus on the central area in each patch

Table S3 Dice scores and Jaccard indices for automatic segmentation models in training, testing and validation sets

	**Dice score**	**Jaccard index**
Training (C1-IBD)	0.947 ± 0.072	0.907 ± 0.108
Testing (C1-IBD)	0.874 ± 0.058	0.780 ± 0.082
Testing (C1-Controls)	0.859 ± 0.075	0.760 ± 0.104
Validation (C2)	0.941 ± 0.093	0.901 ± 0.149
Validation (C3)	0.944 ± 0.061	0.901 ± 0.107

IBD: inflammatory bowel disease

Table S4 Repeatability test based on 30 randomly sampled cases

	**Semi-automatic**	**U-net**	**U-net+Manual adjustment**	**P1***	**P2***
Dice score	0.91 (0.83, 0.93)	0.89 (0.84, 1.00)	0.91 (0.84, 1.00)	0.68	0.71
Jaccard index	0.83 (0.71, 0.87)	0.81 (0.73, 1.00)	0.83 (0.73, 0.99)	0.68	0.71
Time (minutes)	34.50 ± 5.01	1.14 ± 0.32	5.22 ± 2.08	**<0.001**	**<0.001**

P1: Semi-automatic and U-net, P2: Semi-automatic and U-net+Manual adjustment.

Table S5 Comparison of visceral adipose indexes at different levels in three groups

Levels	CD group (n=365)	UC group (n=241)	Control group (n=166)	P1*	P2*	P3*
L1-1, × 10^2 cm^2/m^2	0.113±0.116	0.195±0.205	0.236±0.2262	**<0.001**	**<0.001**	0.11
L1-2	0.124±0.122	0.204±0.208	0.251±0.226	**<0.001**	**<0.001**	0.09
L1-3	0.133±0.128	0.214±0.208	0.264±0.229	**<0.001**	**<0.001**	0.09
L1-4	0.140±0.132	0.221±0.208	0.277±0.234	**<0.001**	**<0.001**	0.06
L1-5	0.151±0.138	0.229±0.210	0.292±0.245	**<0.001**	**<0.001**	**0.04**
L1-L2	0.155±0.141	0.234±0.212	0.298±0.250	**<0.001**	**<0.001**	**0.04**
L2-1	0.159±0.144	0.236±0.209	0.303±0.252	**<0.001**	**<0.001**	**0.03**
L2-2	0.167±0.146	0.241±0.210	0.313±0.254	**<0.001**	**<0.001**	**0.02**
L2-3	0.172±0.149	0.244±0.210	0.317±0.256	**<0.001**	**<0.001**	**0.02**
L2-4	0.176±0.148	0.244±0.208	0.319±0.250	**<0.001**	**<0.001**	**0.01**
L2-5	0.182±0.149	0.246±0.207	0.323±0.250	**<0.001**	**<0.001**	**0.01**
L2-L3	0.185±0.149	0.246±0.206	0.322±0.248	**<0.001**	**<0.001**	**0.01**
L3-1	0.186±0.151	0.246±0.205	0.324±0.249	**<0.001**	**<0.001**	**0.009**
L3-2	0.189±0.154	0.246±0.204	0.325±0.250	**0.002**	**<0.001**	**0.008**
L3-3	0.189±0.154	0.244±0.202	0.321±0.249	**0.003**	**<0.001**	**0.009**
L3-4	0.193±0.152	0.244±0.200	0.323±0.244	**0.007**	**<0.001**	**0.007**
L3-5	0.198±0.149	0.242±0.195	0.325±0.241	**0.03**	**<0.001**	**0.003**
L3-L4	0.200±0.148	0.238±0.192	0.320±0.235	0.10	**<0.001**	**0.002**
L4-1	0.201±0.148	0.234±0.187	0.314±0.225	0.22	**<0.001**	**0.002**
L4-2	0.201±0.147	0.231±0.182	0.310±0.216	0.29	**<0.001**	**0.002**
L4-3	0.200±0.144	0.228±0.176	0.307±0.206	0.33	**<0.001**	**<0.001**
L4-4	0.204±0.139	0.227±0.169	0.310±0.199	0.54	**<0.001**	**<0.001**
L4-5	0.204±0.133	0.223±0.160	0.306±0.191	070	**<0.001**	**<0.001**
L4-L5	0.201±0.128	0.216±0.152	0.297±0.184	0.85	**<0.001**	**<0.001**
L5-1	0.199±0.123	0.209±0.142	0.290±0.173	0.95	**<0.001**	**<0.001**
L5-2	0.198±0.120	0.206±0.136	0.286±0.166	0.97	**<0.001**	**<0.001**
L5-3	0.196±0.114	0.203±0.131	0.287±0.160	0.97	**<0.001**	**<0.001**
L5-4	0.193±0.112	0.197±0.128	0.283±0.152	0.97	**<0.001**	**<0.001**
L5-5	0.183±0.108	0.188±0.123	0.272±0.145	0.97	**<0.001**	**<0.001**
L5-Pelvis	0.175±0.103	0.180±0.119	0.256±0.138	0.97	**<0.001**	**<0.001**
Pelvis-1	0.167±0.098	0.172±0.115	0.245±0.131	0.97	**<0.001**	**<0.001**
Pelvis-2	0.163±0.092	0.173±0.112	0.256±0.136	0.88	**<0.001**	**<0.001**
Pelvis-3	0.166±0.085	0.185±0.111	0.256±0.129	0.22	**<0.001**	**<0.001**
Pelvis-4	0.140±0.074	0.169±0.169	0.211±0.103	**0.001**	**<0.001**	**0.001**
Pelvis-5	0.035±0.035	0.051±0.068	0.045±0.049	**0.004**	**0.01**	0.33

*P1: CD group and UC group, P2: CD group and control group, P3: UC group and control group.

Table S6 Comparison of visceral adipose tissue ratio at different levels in three groups

Levels	CD group (n=365)	UC group (n=241)	Control group (n=166)	P1*	P2*	P3*
L1-1, %	0.368±0.400	0.593±0.670	0.408±0.492	**<0.001**	0.99	**0.049**
L1-2	0.404±0.424	0.624±0.678	0.436±0.501	**<0.001**	0.99	**0.049**
L1-3	0.438±0.450	0.657±0.685	0.454±0.500	**<0.001**	0.99	**0.03**
L1-4	0.463±0.466	0.681±0.695	0.478±0.505	**<0.001**	0.99	**0.04**
L1-5	0.502±0.507	0.708±0.701	0.502±0.506	**<0.001**	0.99	**0.03**
L1-L2	0.519±0.519	0.731±0.722	0.518±0.519	**<0.001**	0.99	**0.03**
L2-1	0.529±0.531	0.744±0.719	0.521±0.513	**<0.001**	0.99	**0.02**
L2-2	0.558±0.549	0.767±0.728	0.542±0.522	**0.002**	0.99	**0.02**
L2-3	0.575±0.561	0.774±0.714	0.558±0.547	**0.004**	0.99	**0.03**
L2-4	0.584±0.550	0.765±0.688	0.565±0.539	**0.009**	0.99	**0.046**
L2-5	0.612±0.567	0.774±0.683	0.575±0.549	**0.04**	0.99	**0.047**
L2-L3	0.626±0.587	0.770±0.681	0.574±0.542	0.13	0.99	**0.049**
L3-1	0.630±0.594	0.764±0.673	0.571±0.536	0.17	0.99	**0.049**
L3-2	0.631±0.606	0.757±0.651	0.569±0.532	0.25	0.99	**0.049**
L3-3	0.639±0.590	0.749±0.638	0.565±0.544	0.36	0.98	**0.049**
L3-4	0.673±0.614	0.748±0.627	0.576±0.551	0.43	0.99	0.07
L3-5	0.686±0.626	0.750±0.618	0.594±0.572	0.91	0.95	0.10
L3-L4	0.695±0.635	0.750±0.628	0.595±0.590	0.98	0.89	0.11
L4-1	0.695±0.642	0.746±0.632	0.583±0.567	0.99	0.66	0.10
L4-2	0.701±0.647	0.739±0.621	0.575±0.541	0.99	0.54	0.09
L4-3	0.725±0.645	0.739±0.617	0.573±0.525	0.99	0.45	0.08
L4-4	0.746±0.683	0.748±0.616	0.589±0.535	0.99	0.37	0.10
L4-5	0.745±0.681	0.751±0.622	0.591±0.539	0.99	0.24	0.10
L4-L5	0.754±0.698	0.731±0.600	0.578±0.539	0.99	0.16	0.10
L5-1	0.754±0.698	0.718±0.585	0.574±0.548	0.99	0.11	0.11
L5-2	0.754±0.703	0.713±0.579	0.572±0.549	0.99	0.11	0.11
L5-3	0.761±0.702	0.718±0.591	0.585±0.566	0.99	0.14	0.13
L5-4	0.752±0.696	0.708±0.599	0.587±0.573	0.99	0.21	0.19
L5-5	0.722±0.697	0.671±0.585	0.564±0.539	0.99	0.24	0.20
L5-Pelvis	0.682±0.647	0.627±0.534	0.530±0.509	0.99	0.21	0.20
Pelvis-1	0.636±0.587	0.593±0.512	0.497±0.461	0.99	0.20	0.20
Pelvis-2	0.618±0.566	0.589±0.492	0.505±0.430	0.99	0.42	0.20
Pelvis-3	0.664±0.618	0.703±0.655	0.522±0.455	0.99	0.21	**0.049**
Pelvis-4	0.576±0.573	0.688±0.657	0.464±0.489	0.40	0.48	**0.007**
Pelvis-5	0.175±0.289	0.248±0.429	0.126±0.255	0.24	0.70	**0.03**

*P1: CD group and UC group, P2: CD group and control group, P3: UC group and control group.

Table S7 Binary logistic regression analysis of clinical and imaging for differentiation between Crohn's disease and ulcerative colitis

	Univariate			Multivariate		
	**OR**	**95%CI**	***p* value**	**OR**	**95%CI**	***p* value**
Age	0.94	0.93 - 0.95	<0.001	0.94	0.93 - 0.96	<0.001
Sex	0.58	0.41 - 0.81	0.002	0.87	0.55 - 1.38	0.56
Perianal disease	7.30	4.31 - 12.38	<0.001	5.61	3.00 - 10.50	<0.001
Alb	1.00	0.99 - 1.01	0.79	-	-	-
CRP	1.01	1.01 - 1.02	<0.001	1.01	1.00 - 1.01	0.04
ESR	1.02	1.01 - 1.02	<0.001	1.01	1.00 - 1.02	0.02
CVmodel	7.02	1.94 - 25.4	0.003	6.05	1.17 - 31.12	0.03

Alb: serum albumin, CRP: C-reaction protein, ESR: erythrocyte sedimentation rate, CV: Coefficient of variability, OR: Odds ratios, CI: Confidence intervals.
